# Evaluation of *Healthy South Texas Asthma Program* on improving health outcomes and reducing health disparities among the underserved Hispanic population: using the RE-AIM model

**DOI:** 10.1186/s12887-021-02991-8

**Published:** 2021-11-16

**Authors:** Genny Carrillo, Taehyun Roh, Juha Baek, Betty Chong-Menard, Marcia Ory

**Affiliations:** 1 Department of Environmental and Occupational Health, School of Public Health, Texas A&M University, 212 Adriance Lab Road, College Station, TX 77843 USA; 2 Department of Epidemiology and Biostatistics, School of Public Health, Texas A&M University, 212 Adriance Lab Road, College Station, TX 77843 USA; 3 Postdoctoral Fellow, Center for Outcomes Research, Houston Methodist, 7550 Greenbriar Dr, Houston, TX 77030 USA; 4 Dr. Ramiro R. Casso NAH Campus, South Texas College, 1101 E. Vermont, McAllen, TX 78503 USA

**Keywords:** Asthma education program, Childhood asthma, Hispanic, RE-AIM framework, Health outcomes, Health disparities, South Texas

## Abstract

**Background:**

In the United States, childhood asthma prevalence is higher among low-income communities and Hispanic populations. Previous studies found that asthma education could improve health and quality of life, especially in vulnerable populations lacking healthcare access. This study aims to describe *Healthy South Texas Asthma Program (HSTAP),* an evidence-based asthma education and environmental modification program in South Texas, and evaluate its associations with health-related outcomes among Hispanic children with asthma and their families.

**Methods:**

The RE-AIM (Reach, Effectiveness, Adoption, Implementation, Maintenance) planning and evaluation framework was used as an overarching tool to evaluate the impact of the HSTAP. This educational program included 451 children with asthma and their families living in South Texas, an impoverished area at the Texas-Mexico border. The program consisted of (a) the asthma education (2-h) for children with asthma provided by Respiratory Therapy students at the children’s schools and (b) the home visit *Asthma and Healthy Homes* education and walk-through sessions (at baseline and 3 months) for parents and two follow-up visits (6 and 9–12 months later) led by community health workers. The education was provided in either English or Spanish between September 2015 and August 2020 as part of the Healthy South Texas Initiative. A pre-and post-test design was implemented to assess the differences in health outcomes, knowledge, and behaviors using standardized self-reported surveys as reported by parents. Analyses included primary descriptive analyses, generalized estimating equation models, the Wilcoxon signed-rank test, and the McNemar test.

**Results:**

The HSTAP was significantly associated with improved individual-level outcomes on the frequency of asthma-related respiratory symptoms, including shortness of breath, chest tightness, coughing, and sleep difficulty, among children with asthma, as well as an enhanced asthma knowledge in their family. This study also showed significant associations with children’s school attendance and participation in physical activities and family social events and decreased families’ worry about their asthma management.

**Conclusions:**

The RE-AIM model was a helpful framework to assess the HSTAP on all its components. The results suggest that participation in an asthma education and environmental modification program was associated with improved individual-level health conditions and reduced health disparities among children with asthma in low-income communities.

**Supplementary Information:**

The online version contains supplementary material available at 10.1186/s12887-021-02991-8.

## Background

Chronic diseases are the leading causes of death and disability worldwide and currently account for almost 60% of all deaths and 43% of the global disease burden. Chronic disease conditions are accelerating globally, advancing across every region and pervading all socioeconomic classes [1]. Chronic health diseases such as asthma are more prevalent among low-income and ethnic/minority populations with a lack of medical insurance and limited healthcare access [2]. According to the Centers for Disease Control and Prevention (CDC), the asthma prevalence rate in the United States (U.S.) was 7.7% in adults and 7.5% in children in 2018 [3]. Regional statistics highlight health disparities by location and population group. For example, while approximately 7% of children had asthma in Texas, the annual age-adjusted hospitalization rate among children in the Health Service Region (HSR) 11 is 9.2 per 10,000 [4]. In particular, Hidalgo County, which is included in the Texas HSR 11, has an asthma age-adjusted hospitalization rate of 13.4 per 10,000 [5]. Our previous study showed the prevalence rates of asthma were estimated as 9.4% in all children and 16.7% in children aged 14–18 years between 2014 and 2016 in Hidalgo County, Texas, which has one of Texas’s highest poverty rates [6, 7].

Previous research has demonstrated that asthma educational programs improve knowledge about asthma, self-efficacy in management, and exposure to asthma triggers among families with a child with asthma. In addition, parents learn the importance of medication adherence and enhancing their health behaviors to decrease their child’s asthma attacks, hospitalizations, and emergency room visits [8–10]. However, previous studies are primarily based on small clinic-based populations. There is still a gap in what is known about the impact of asthma education and environmental modification on Hispanic children and their families, especially those living in disadvantaged areas of the Texas-Mexico border.

In 2015, the Texas A&M Health Science Center and the Texas A&M AgriLife Extension System established a partnership to address chronic and infectious diseases in South Texas. The State of Texas provided financial resources through a legislative mandate to reduce the health disparities in South Texas and improve health status and quality of life for the residents in such areas [11]. Our *Healthy South Texas Asthma Program* (HSTAP) drew upon the Program on Asthma Research and Education (PARE), hosted at the Texas A&M University (TAMU) School of Public Health. PARE was a continuation of the McAllen Asthma Coalition developed in 2007 by the TAMU McAllen Campus. The asthma education and environmental modification program has been offered to the Hispanic population living in South Texas’s low-income communities with numerous regional entities for the last 10 years. This study examines the program’s implementation during 2015–2020 using the RE-AIM (Reach, Effectiveness, Adoption, Implementation, and Maintenance) Planning and Evaluation framework [12], including program process evaluation components and individual-level health outcomes and quality of life.

## Materials and methods

### RE-AIM framework

The RE-AIM framework was developed to assess generalizable and evidence-based public health interventions and determine the impact of a program or policy on population health [13]. This framework consists of five elements related to behavioral health interventions: Reach, Effectiveness, Adoption, Implementation, and Maintenance. The RE-AIM model has been widely used in various research areas, including public health, health behavior change, and individual, clinical, organizational, and community settings [12]. For example, a recent study applied the RE-AIM model to present the process and outcomes of a diabetes education program implemented in South Texas [14]. Another research revealed that RE-AIM could be an effective model for planning and assessing clinical and community-based projects [15]. This framework has been applied for evaluating grant applications at health and medical research institutions in the U.S. [16]

This study used five elements of the RE-AIM framework to describe the evidence-based HSTAP and examine its implementation, including program processes and its association with individual-level health outcomes among Hispanic populations in South Texas. Recent literature emphasizes that there may not be comprehensive measurements for all components in community-based studies [17].

### Population settings and targets

The *HSTAP* was implemented to the families of children with asthma in Hidalgo County, Texas (a US-Mexico border region), including urban and rural areas, to address a high number of hospitalizations and a high disease prevalence in this region. In 2019, this county’s total population was 868,707, and 30.3% did not have medical insurance. The median household income is $40,014, lower than Texas’s median ($67,444) [17]. The county’s poverty rate is 26.9, and 18.7% have a bachelor’s degree or higher. About 83.4% of the population speak Spanish at home, and 92.2% are Hispanic or Latino [17]. This study targeted low-income families having children diagnosed with asthma and their parents. The study was reviewed and approved by the Institutional Review Board of Texas A&M University IRB2015–0472. All participant’s parents or legal guardians signed the informed consent forms for the study. All methods were performed following the relevant guidelines and regulations of Texas A&M University.

### Recruitment

The research team primarily recruited participants through elementary and middle schools in the study region. The lead author approached and met with four Superintendents of Independent School Districts (ISD) to seek their participation. After explaining the study and their participation, they agreed that the school nurse could be contacted to help identify children aged 4–18 years old diagnosed with asthma by their healthcare provider. Once identified and before referring any children, the school nurse checked if parents were interested in participating in the study. The study staff provided the school nurse the consent form to obtain the parent’s signature if they agreed. After the parents signed the informed consent form, a Community Health Worker (CHW) contacted parents to provide them with more information about the research and respond to any questions that they might have. A total of 494 families were initially consented and enrolled in the study; however, with withdrawals, HSTAP actively engaged 451 participants until the 3rd household visit for the follow-up health surveys and then 447 participants until the 4th visit for the assessment of environmental and behavioral change.

### Intervention

The *Asthma and Healthy Homes* curriculum for the HSTAP is bilingual in English or Spanish. It comprises tailored information from the curriculum validated by the American Lung Association and the Healthy Homes curriculum developed by the Department of Housing and Urban Development (HUD) [18, 19]. This curriculum, provided to the parents of children with asthma, consists of two sections: one specific to asthma and the other on healthy homes. The *Asthma section* focuses on signs and symptoms of asthma, management of the disease, identification of common triggers, acceptable use of asthma medications, actions to take in case of an asthma attack, and fundamental components of an asthma action plan [19]. The *Healthy Homes section* includes keeping a home dry, clean, ventilated, pest-free, safe, and contaminant-free to improve the indoor environment and decrease hazardous exposures within the home [20].

Since PARE’s inception, the South Texas College Respiratory Department has collaborated providing respiratory therapy (RT) students’ involvement in the asthma education of elementary and middle school students. First, the RT’s were trained with asthma and healthy homes curriculum by the lead author, and as part of the training, the RTs students had a mock-up presentation to practice how to create rapport with participating children. Then, at a later date, the RT’s educate the participating children at their affiliated schools for 2-h.

The children’s parents received four visits of CHWs at their households to provide asthma education using the asthma curriculum. During the first visit (baseline), the CHWs conducted surveys of parents and provided them with an allergen-proof mattress and pillow encasing. Also, they offered a 2-h in-person *Asthma and Healthy Homes* education in the parent’s households. Next, when the CHWs revisited the household 3 months later (the second visit), they offered a home walk-through to identify environmental asthma triggers with the parents and deliver home environmental modification strategies. At the third visit (6 months later), the CHWs administered follow-up surveys and discussed maintaining asthma triggers prevention in the household with parents. At the fourth visit (9–12 months later), the CHWs confirmed the family’s changes to decrease asthma triggers and addressed questions regarding the program.

A manual was developed for the HSTAP program. After receiving three continuing education units (CEUs) for training on the curriculum accredited by the Texas Department of State and Health Services, the CHWs provided education for parents. The CHW’s training followed strict guidelines to apply surveys, curriculum, and all other steps needed to ensure adherence to standard operating procedures and maintain assessment fidelity.

### Data collection

The study population included participants enrolled in this state-funded HSTAP for 5 years, from September 2015 to August 2020. Data were collected three times (at baseline, 6 months, and 9–12 months) during the household visits. The surveys were applied at the first visit to collect baseline information before the intervention to assess the health outcomes and quality of life in asthmatic children and their families. The follow-up surveys were applied at 6 months during the third visit. The Children’s Health Survey for Asthma (CHSA), developed by the American Academy of Pediatrics, was administered to the parents to collect information on asthma symptoms, physical activity, and emotional health for the child and family [21]. The CHSA was replaced by the Asthma Pediatric Quality of Life (PedsQL) in 2018, which measures asthma-specific health-related quality of life in asthmatic children. Other surveys developed for the research team administered to the parents included a) Asthma and Healthy Homes test (to measure knowledge); b) Asthma Home Environment and Triggers Checklist (to identify asthma triggers at home); c) Behavioral Changes Survey (applied at 9–12 months to measure the behavioral changes that the parent did to improve the exposure of asthma triggers), and d) Post-training Satisfaction Assessment applied at the end of the study (9–12 months) [9].

### Data analysis

Descriptive analyses were conducted to describe the study participants’ characteristics and home environments, including the mean and standard deviation (SD). The frequency of health outcomes, behavior, and asthma severity reported by parents was measured with a Likert scale (e.g., scores of 1–5 for never, almost never, sometimes, often, and almost always each) before and after the intervention. For each survey, the scores were scaled to have a range of 0–100 to make scores comparable over different items. The higher scores indicated better outcomes (less frequency of symptoms and limitations), and total scores were computed by averaging scores for each item. For PedsQL, each section’s scores were calculated by averaging each item, and total scores were the average of each section. The scores were treated as continuous variables to test a statistical significance of improvements, representing the degree of frequency and severity. The generalized estimating equation models were fitted to assess the improvement of children’s health outcomes, asthma severity, behavior, emotional health of their families, and knowledge of asthma after the intervention, with adjustment for baseline score, gender, age, and previous asthma education (yes/no). Children’s PedsQL data were analyzed using the Wilcoxon signed-rank test due to the small sample size (*n* = 14). In addition, the McNemar test was conducted to assess the difference in proportions of correct answers between the baseline and follow-up knowledge for each question. A *p*-value less than 0.05 was considered statistically significant. All statistical analyses were conducted by using SAS version 9.4 [22].

## Results

### Reach

Reach refers to “the absolute number, proportion, and representativeness of individuals who are willing to participate in a given initiative, intervention, or program, and reasons why or why not” [23]. The school nurses from the targeted ISD’s were trained about the signs and symptoms of asthma by the lead author to identify a student with asthma symptoms. The school nurses discussed the study with participating parents after they agreed to participate and signed the consent, and at a later date, asthma staff retrieved it. Furthermore, a community awareness campaign was undertaken to make educational information about asthma widely available to raise awareness about childhood asthma. The asthma campaign involved participating in school health fairs, library events, and back-to-school events reaching over 10,000 individuals who attended those events during the study.

Table 1 presents the demographic characteristics of 451 participants in the asthma educational intervention between 2015 and 2020. The majority of the participants (91.1%) were mothers of children with asthma, with the largest percentage of the children being boys (58.3%) and those aged between 4 and 11 years (69.8%). Most parents and their children were classified as Hispanic (97.1 and 99.1%, respectively), and 65% of participants had never previously participated in the asthma education program. Of the 494 parents approached to participate, 91% accepted to enroll and completed the study (451 participants).

**Table 1:** Demographic Characteristics of the *HSTAP* Participants (*N* = 451, 2015–2020)

Characteristics	N	(%)
**Parents’ Gender**		
Male	40	(8.9%)
Female	411	(91.1%)
**Parents’ Ethnicity**		
Hispanic	438	(97.1%)
Non-Hispanic	13	(2.9%)
**Children’s Ethnicity**		
Hispanic	447	(99.1%)
Non-Hispanic	4	(0.9%)
**Children’s Gender**		
Boys	263	(58.3%)
Girls	188	(41.7%)
**Children’s Age**		
< 4	33	(7.3%)
4–11	315	(69.8%)
12–19	102	(22.6%)
**Previous Asthma Education**		
Yes	109	(35%)
No	342	(65%)

### Effectiveness

Effectiveness is defined as “the impact of an intervention on important individual outcomes, including potential negative effects, and broader impact including quality of life and economic outcomes; and variability across subgroups (generalizability or heterogeneity of effects)” [23]. This study assessed whether the asthma education was associated with improvements in the health outcomes and quality of life for children with asthma and their families. Major focal areas were asthma symptoms, activities performed by the child and family, and their emotional well-being.

In 2015–2018, the frequencies of the asthma symptoms, limitations of school and family activities, and families’ emotional health were surveyed in 351 participants during the first visit and their follow-up at 6 months. Table 2 summarizes the scores in each category at baseline before the intervention and at 6-month follow-up after implementing the intervention, which was evaluated using the CHSA. We found a significant improvement (*p* < 0.01) in all asthma-related symptoms, including shortness of breath, chest tightness, wheezing with/without a cold, coughing, and difficulty sleeping due to symptoms. School and family activities’ limitations were significantly decreased (*p* < 0.05) in all categories, including school gym participation, sports activities or running, family social plan, outdoor activities, loss of sleep, and school absence. In addition, the emotional health in families with asthmatic children was significantly improved (*p* < 0.05) after asthma education. Parents showed fewer doubts about therapy, more confidence in handling their child’s asthma attack, a greater hope that their child will get better, and less worry about the child’s medical cost for asthma.

**Table 2:** Average Scores (SD) of Asthma-Related Symptoms, Limitations of School and Family Activities, and Families’ Emotional Health at Baseline and 6-Month Follow-up of the HSTAP from CHSA in parents between 2015 and 2018 (*n* = 351)

CHSA Items	Baseline	Follow-up (6 months)	***p***-value
**Asthma-Related Symptoms**			
Shortness of breath	79.9 (21.4)	91.7 (15.0)	< 0.01^*^
Chest tightness	84.4 (21.6)	93.4 (13.4)	< 0.01^*^
Wheezing without a cold	82.9 (22.7)	91.6 (15.6)	< 0.01^*^
Wheezing with a cold	75.8 (27.1)	93.3 (14.4)	< 0.01^*^
Coughing	64.7 (27.4)	80.5 (20.3)	< 0.01^*^
Difficulty sleeping due to symptom	72.4 (28.8)	91.1 (17.5)	< 0.01^*^
**Limitation of School and Family Activities**			
School gym participation	89.5 (20.3)	93.8 (13.9)	< 0.01^*^
Sports and running	88.3 (22.0)	93.3 (14.3)	< 0.01^*^
Family social plan	89.5 (20.6)	91.6 (18.8)	0.02^*^
Outdoor activities	81.2 (26.8)	91.4 (18.9)	< 0.01^*^
Loss of sleep	91.2 (15.0)	94.0 (12.7)	< 0.01^*^
Missing school or work	96.1 (11.0)	97.1 (10.2)	0.04^*^
**Families’ Emotional Health**			
Doubts about therapy	64.4 (28.0)	70.2 (24.2)	< 0.01^*^
Not confident in handling asthma attack	57.5 (27.5)	60.8 (23.1)	0.04^*^
Losing hope of getting better	74.0 (27.8)	83.9 (20.4)	< 0.01^*^
Worry about medical cost	69.8 (29.0)	78.3 (26.8)	< 0.01^*^
Worry about care quality	72.8 (28.0)	82.5 (24.9)	< 0.01^*^
Worry about social isolation	61.4 (31.9)	70.6 (30.1)	< 0.01^*^

Note: A higher score means improvement

^*^Statistically significant improvement (*p* < 0.05)

In 2018–2019, and 2020, the asthma-related quality of life was evaluated using PedsQL in both parents and children, including asthma symptoms, treatment, worry, and communication at baseline and 6-month follow-up (Table 3). The average scores of symptoms, treatment, concern, and communication in parents increased significantly between baseline and 6-month follow-up, indicating significant improvements in all categories after the HSTAP educational sessions (*p* < 0.05). In children, the problems with symptoms and treatment were improved considerably (*p* < 0.05), while the problems with worry and communication showed a non-significant change.

**Table 3:** Average Scores (SD) of Asthma-Related Quality of Life in Parents and Children with Asthma at Baseline and 6-Month Follow-up of the HSTAP from PedsQL in parents and children between 2018 and 2020 (*n* = 100)

PedsQL Items	Baseline	Follow-up (6 months)	***p***-value
**Parents**			
Problems with symptoms	66.6 (17.5)	84.8 (12.2)	< 0.01^*^
Problems with treatment	86.3 (12.1)	94.1 (8.75)	< 0.01^*^
Problems with worry	66.5 (29.6)	77.1 (28.8)	< 0.01^*^
Problems with communication	89.6 (21.4)	97.3 (9.17)	< 0.01^*^
**Children**			
Problems with symptoms	89.5 (20.3)	93.8 (13.9)	0.02^*^
Problems with treatment	88.3 (22.0)	93.3 (14.3)	< 0.01^*^
Problems with worry	89.5 (20.6)	91.6 (18.8)	0.21
Problems with communication	81.2 (26.8)	91.4 (18.9)	0.28

Note: A higher score means improvement

*Statistically significant improvement (*p* < 0.05)

The average scores of asthma knowledge in 447 participants between 2015 and 2020 were 67.8 (SD 19.0) at baseline and 77.0 (SD 19.6) (out of 100) at 6 months after HSTAP education sessions, showing a statistically significant increase after adjusting for baseline scores (*p* < 0.01). Table 4 shows the changes in the proportion of correct answers for each item between baseline and follow-up. Among 10 items, statistically significant improvement was found in participants’ knowledge in 7 items: 7 principles of a Healthy Home to maintain the home (*p* < 0.01); Dust mites live in stuffed toys (*p* < 0.01); Secondhand smoke is directly linked to asthma (*p* = 0.01); the best way to safely reduce or eliminate pests at home (*p* < 0.01); Symptoms of asthma (*p* = 0.04); Sign that child’s asthma is controlled includes not needing to go to the emergency room (*p* < 0.01); Frass is defined as rodent droppings (*p* < 0.01).

**Table 4:** Average Scores (SD) of Parents’ Asthma Knowledge Test and Percentage of Correct Responses to Individual Questions at Baseline and 6-Month Follow-up of the HSTAP (*n* = 447)

Question	% Correct Response		***p***-value
	Baseline	Follow-up (6 months)	
The seven principles of a Healthy Home to maintain the home	84.0	92.3	< 0.01^*^
Dust mites live in dust toys	70.7	85.1	< 0.01^*^
Secondhand smoke is directly linked to asthma	89.4	93.3	0.01^*^
What is the best way to safely reduce or eliminate pests in your home?	84.3	93.8	< 0.01^*^
Indoor pollutants that can trigger asthma include	92.1	90.3	0.86
What statement is true: most asthma episodes can be prevented	36.0	39.0	0.09
Which is a symptom of asthma	29.8	33.0	0.04*
A sign that your child’s asthma is controlled includes not needing to go to the emergency room	49.9	70.9	< 0.01^*^
Frass is defined as rodent droppings	37.6	76.9	< 0.01^*^
What is the best behavior to follow in your household?	95.5	96.4	0.25
**Total Score (max 100)**	67.8 (19.0)	77.0 (19.6)	< 0.01*

^*^Statistically significant improvement at *p* < 0.05, after adjusting for baseline score

We observed that the household environments and behaviors were changed and improved in 86.4% of participants between 9 and 12 months after the HSTAP intervention during the 4th visit (Supplementary Table 1). The top 5 items changed by the participants were: keeping home free of clutter (62.4%), not allowing trash to accumulate at home (58.4%), not allowing smoking at home (55.7%), limiting the number of plush toys at home (52.6%), and monitoring and changing air conditioner filters frequently (50.1%). The lowest implemented changes included the repairs of holes in the walls (13.9%), plumbing leaks (8.9%), and peeling or chipped paint at home (3.6%).

### Adoption

Adoption is “the absolute number, proportion, and representativeness of settings and intervention agents (people who deliver the program) who are willing to initiate a program, and why” [23]. The Program on Asthma Research and Education (PARE), which initiated the Healthy South Texas Asthma Program (HSTAP), served as a continuation of the McAllen Asthma Coalition developed by the TAMU McAllen Campus to provide the HSTAP to a population with asthma in disadvantaged communities of South Texas. All the Independent School Districts and schools we approached allowed us to implement the HSTAP with their students with the asthma diagnosis after their parents were informed about the study and signed consent to be contacted. The HSTAP has moved beyond the original sites, and it has been expanded for the Public Health Region XI by the Driscoll Children’s Hospital in Corpus Christi, Texas. Since the program’s implementation, we have collaborated with regional partners to deliver asthma education and community outreach. The Texas Department of State and Health Services, University of Texas Rio Grande Valley, School of Nursing, South Texas College, Respiratory Therapy Program, local health departments, and many Independent Health Districts have collaborated with PARE continuously since its inception [24].

### Implementation

Implementation refers to “the intervention agents” fidelity to the various elements of an intervention’s key functions or components, including consistency of delivery as intended and the time and cost of the intervention; importantly, it also includes adaptations made to interventions and implementation strategies” [23].

The research team coordinated the training of children by RT students and their parents by CHWs. Their parents responded to several surveys described previously and provided by the CHWs. We measured the implementation levels (i.e., fidelity to delivering the asthma curriculum and surveys throughout the study as the developers intended) in the participant’s households and schools with children. The interviewers went through a checklist of principles for the order of surveys and issues they needed to cover with each participant. The researchers determined how well the CHWs adhered to the program’s principles when implementing the intervention by a designated quality control coordinator to review the delivery process, confirm that they were compliant, and annotate the aspects that needed to be corrected. The research team discussed problem-solving issues monthly to identify challenges the CHWs faced while reaching out to participants and educating them. There were adaptations to the program reviewed with the research team to enhance program reach and retention. At the beginning of the program, the CHWs called participants to schedule the first visit and all others after completing the four planned. The last 9–12 months visit was changed to a phone call for security reasons due to the COVID-19 pandemic in March 2020.

One of the challenges was the length of the CHSA survey, which the PedsQL replaced. Another challenge was that the CHWs sometimes did not have a cellular phone due to a lack of payment support. The CHWs had to visit participants’ households directly to set up the meeting and administer the surveys in such cases. Despite these challenges, our retention rate over the entire study period was approximately 91%.

### Maintenance

Within the RE-AIM framework, maintenance is the “program’s long-term effects on outcomes after a program is completed at the individual level, and the extent to which a program or policy becomes institutionalized or part of the routine organizational practices and policies at the setting level” [23]. We reported positive changes in HSTAP participants’ individual-level health outcomes and quality of life in the effectiveness section. At the setting level, several programs, including this asthma education and environmental modification program, were embraced in the Healthy South Texas initiative from 2015 to 2020 to be housed within and overseen by faculty from TAMU and community partners. While HSTAP experienced 5 years of continuous institutional support, we could not continue to follow-up or enroll new participants after the study ended. Due to a restructuring of the Healthy South Texas initiative, the funding for asthma programming was not renewed by the Texas legislative commitment in its third biennium. Therefore, financing for HSTAP is needed to continue the program that has been in South Texas for over 10 years.

## Discussion

The Healthy South Texas Initiative was created with over 15 million dollars of legislative funding identified in direct support and in-kind dollars from private and public sources. The initiative included a focus on asthma and other prevention activities for other chronic diseases like diabetes. Two hospitals in the public health region 11 adopted the educational curriculum. This study described the Texas A&M HSTAP, providing education and community outreach with environmental modifications to children with asthma and their families in South Texas’s disadvantaged communities. It assessed the program’s benefits and challenges using the general RE-AIM framework, focusing on reach, effectiveness, adoption, implementation, and maintenance as data permitted in this real-world study [16]. Significant positive changes in the individual level were observed in children’s physical health, leading to improved school attendance (as per parental report) and their ability to participate in school physical activities and family social events. Specifically, we found that the HSTAP was associated with significant decreases in the frequency of asthma-related respiratory symptoms, including shortness of breath, chest tightness, coughing, and sleep difficulty, among children with asthma and enhanced asthma knowledge in their families.

Previous studies have found that a home-based education improved health outcomes and home environments among children with asthma and their family [9, 25–28]. However, scarce asthma education programs have focused on the Hispanic population in the Texas-Mexico border area specifically. In HSTAP, almost all of the service region participants were Hispanic population living in South Texas. The HSTAP was led by CHWs, who understand the target populations’ culture and values [29, 30] and are bilingual and certified healthcare workers. The CHWs were an essential factor that allowed the program to reach out to the people and improve their health outcomes and quality of life. Their involvement might have contributed to keeping most participants in the study until the final visit, leading to the high retention rate. (91%).

In addition, the Adoption and Implementation RE-AIM Elements were reflected by adherence to community-based participatory principles [30]. Collaborating with regional partners, such as school nurses and CHWs, was essential for recruiting schools and children with asthma and their parents. An actionable intervention protocol in sequential steps and monthly check-ins with CHWs helped ensure program implementation consistency [23]. Further, a home visit was an effective way to deliver education in a comfortable place and identify asthma triggers at each home [9]. An innovative implementation strategy was linking school and home sectors [12]. Combining free asthma education, a spacer with an allergen-proof mattress, and pillow encasing was a critical element that helped vulnerable populations who may have otherwise had limited access to that service to participate in the program.

As reported in other studies, maintenance at the individual and setting levels was challenging among the RE-AIM elements [31]. Our findings showed that the HSTAP was associated with improved participants’ health outcomes at 6 months and household environment and behaviors at 9–12 months after implementing the intervention. The education program developed with the respiratory therapy students who teach at elementary and middle schools was integrated as part of their clinical rotation in pediatrics. Health programs have difficulty in sustainability due to a lack of funds/grants and resources. However, innovative ways to involve students in the education program and keep them engaged with communities can be done using virtual methods to teach and follow-up participants and collaborate with hospitals to implement prevention programs. Those hospitals would be in a better situation to implement follow-ups in the long term. Although the Healthy South Texas Initiative no longer supports the HSTAP, two hospitals in the public health region 11 adopted the asthma program, suggesting more extended maintenance.

The findings have significant public health implications for persistently providing evidence-based education and environmental change to children with asthma and their families living in disadvantaged communities. The HSTAP can potentially reduce health disparities in access to education and service, enhance health outcomes for children with asthma in South Texas, and improve overall family quality of life.

### Limitation of the study

Despite the many benefits of our HSTAP, this study has several limitations to consider when interpreting the results. First, this study was a real-world evaluation of an asthma education program and did not include any comparison groups; hence potentially confounding factors associated with study outcomes were not assessed. As a result, our findings suggest health benefits related to the intervention, but the results are suggestive versus definitive.

Moreover, the health outcomes are primarily self-reported by parents and their children with asthma. It was not feasible to obtain participants’ clinical records to confirm their hospitalizations and emergency room visits since participants without medical insurance rotate hospitals when they require urgent medical attention. Similarly, impacts on school-related events were reported by parents versus having documented school records. An associated limitation was having all the data collection by CHWs that could have contributed to intervention bias. We also acknowledge the problem of having key asthma impact measures that change throughout the study. We have presented data as available at different time points.

Additionally, there may be an issue of generalization. This study included only a single asthma education program implemented in some communities of South Texas, so the results might not be generalizable to other regions with different population settings.

Lastly, it may be hard to assess the program’s long-term effect on health since the primary outcome was measured at 6-months, not at 12-months, due to logistical issues, which is a limitation because the intervention was not finalized and asthma is seasonal. Thus, a longer-term follow-up of these population’s outcomes after the study period is needed to document the program’s long-term effect. Additionally, having continuous funding support and partnerships with community organizations is a significant challenge for an asthma program’s long-term sustainability toward a low-income Hispanic population.

## Conclusions

This study showed that participation in the HSTAP program was associated with improved individual-level health conditions and reduced health disparity in education among children with asthma in low-income communities. The results suggest that developing and expanding these education programs and environmental modifications are needed to enhance asthma prevention, self-management, and quality of life for children with asthma living in regions with disadvantaged populations. The RE-AIM model was a helpful framework to evaluate this program across all five components.

## Supplementary Information

**Additional file 1: Supplementary Table 1.** Changes in Household Environment and Behaviors Reported by Parents at 9–12-month Follow-up of the HSTAP (*n* = 447).

## Data Availability

The datasets used and/or analyzed during the current study are available from the corresponding author on reasonable request.

## Abbreviations

RE-AIM: Reach, Effectiveness, Adoption, Implementation, Maintenance
HSR: Health Service Region
HSTAP: *Healthy South Texas Asthma Program*
PARE: Program on Asthma Research and Education
ISD: Independent School District
HUD: Department of Housing and Urban Development
CHW: Community Health Worker
CHSA: Children’s Health Survey for Asthma
PedsQL: Asthma Pediatric Quality of Life

## References

[1] Collaborators GRF. GBD 2015 risk factors Collaborators (2016). Global, regional, and national comparative risk assessment of 79 behavioural, environmental and occupational, and metabolic risks or clusters of risks, 1990–2015: a systematic analysis for the global burden of disease study 2015. Lancet..

[2] Price JH, Khubchandani J, McKinney M, Braun R (2013). Racial/ethnic disparities in chronic diseases of youths and access to health care in the United States. Biomed Res Int.

[3] Centers for Disease Control and Prevention (2019). Asthma Surveillance Data.

[4] Texas Department of State Health Services (2016). Texas Childhood Asthma Fact Sheet.

[5] Carrillo G, Han D, Lucio RL, Seol YH, Chong-Menard B, Smith K (2015). Impacting environmental and public health through the use of dual targeted and tailored asthma educational interventions. J Environ Public Health.

[6] Carrillo G, Perez Patron MJ, Johnson N, Zhong Y, Lucio R, Xu X (2018). Asthma prevalence and school-related hazardous air pollutants in the US-México border area. Environ Res.

[7] Ura A (2016). Latest Census data shows poverty rate highest at border, Lowest in Suburbs.

[8] Clark N, Starr NS (1994). Management of Asthma by patients and families. Am J Respir Crit Care Med.

[9] Carrillo G, Spence-Almaguer E, Lucio RL, Chong-Menard B, Smith K (2015). Improving asthma in Hispanic families through a home-based educational intervention. Pediatr Allergy Immunol Pulmonol.

[10] Crocker DD, Kinyota S, Dumitru GG, Ligon CB, Herman EJ, Ferdinands JM (2011). Effectiveness of home-based, multi-trigger, multicomponent interventions with an environmental focus for reducing asthma morbidity: a community guide systematic review. Am J Prev Med.

[11] Healthy South Texas. Bi-annual Report 2016–2017. https://agrilife.org/healthytexas/files/2018/02/WEB-HSTX-33282-0218_Bi-annual_report.pdf.

[12] Glasgow RE, Harden SM, Gaglio B, Rabin B, Smith ML, Porter GC (2019). RE-AIM Planning and Evaluation Framework: Adapting to New Science and Practice With a 20-Year Review. Front Public Health.

[13] Glasgow REVT, Boles SM (1999). Evaluating the public health impact of health promotion interventions: the RE-AIM framework. Am J Public Health.

[14] Ory MGLS, Towne SD, Flores S, Gabriel O, Smith ML (2020). Implementing a Diabetes Education Program to Reduce Health Disparities in South Texas: Application of the RE-AIM Framework for Planning and Evaluation. Int J Environ Re Public Health.

[15] Kwan BM, McGinnes H, Ory MG, Estabrooks PA, Waxmonsky JA, Glasgow RE (2019). RE-AIM in the Real World: Use of the RE-AIM Framework for Program Planning and Evaluation in Clinical and Community Settings. Front Public Health.

[16] Shoup JA, Gaglio B, Varda D, Glasgow RE (2015). Network analysis of RE-AIM framework: chronology of the field and the connectivity of its contributors. Transl Behav Med.

[17] Census Bureau US (2019). American community survey 1-year estimates Census reporter profile page for Hidalgo County, TX.

[18] U.S. Department of Housing and Urban Development. Healthy Homes. https://www.hud.gov/program_offices/healthy_homes/hhi.

[19] Zuniga GC, Kirk S, Mier N, Garza NI, Lucio RL, Zuniga MA (2012). The impact of asthma health education for parents of children attending head start centers. J Community Health.

[20] Neltner T (2010). National healthy homes training center and network: building capacity for healthy homes. J Public Health Manag Pract.

[21] American Academy of Pediatrics (2008). User’s Guide: Children’s Health Survey for Asthma (CHSA).

[22] SAS Institute Inc. SAS/ACCESS® 9.4 Interface to ADABAS: Reference Cary, NC: SAS 2013. https://communities.sas.com/t5/Statistical-Procedures/SAS-Citation-quot-EXAMPLE.

[23] Gaglio B, Shoup JA, Glasgow RE (2013). The RE-AIM framework: a systematic review of use over time. Am J Public Health.

[24] Zuniga GC, Hernandez T, Kirk S, Nadeau N, Chong-Menard B, Lucio RL (2011). A multi-institutional collaboration to develop asthma education for school settings in South Texas. Public Health Rep.

[25] Baek J, Huang K, Conner L, Tapangan N, Xu X, Carrillo G (2019). Effects of the home-based educational intervention on health outcomes among primarily Hispanic children with asthma: a quasi-experimental study. BMC Public Health.

[26] Krieger JW, Takaro TK, Song L, Weaver M (2005). The Seattle-King County healthy homes project: a randomized, controlled trial of a community health worker intervention to decrease exposure to indoor asthma triggers. Am J Public Health.

[27] Cherrington A, Ayala G, Amick H, Scarinci I, Allison J, Corbie-Smith G (2008). Applying the community health worker model to diabetes management: using mixed methods to assess implementation and effectiveness. J Health Care Poor Underserved.

[28] Campbell JD, Brooks M, Hosokawa P, Robinson J, Song L, Krieger J (2015). Community health worker home visits for Medicaid-enrolled children with asthma: effects on asthma outcomes and costs. Am J Public Health.

[29] Islam N, Nadkarni S, Zahn D, Skillman M, Kwon SC, Trinh-Shevrin C (2015). Integrating community health workers within patient protection and affordable care act implementation. J Public Health Manag Pract.

[30] Bopp M, Wilcox S, Laken M, Hooker SP, Saunders R, Parra-Medina D (2007). Using the RE-AIM framework to evaluate a physical activity intervention in churches. Prev Chronic Dis.

[31] Ory MG, Lee S, Towne SD, Flores S, Gabriel O, Smith ML (2020). Implementing a diabetes education program to reduce health disparities in South Texas: application of the RE-AIM framework for planning and evaluation. Int J Environ Res Public Health.

