# Supplementary material for: Evaluation of Healthy South Texas Asthma Program on improving health outcomes and reducing health disparities among the underserved Hispanic population: using the RE-AIM model
Source: BMC Pediatr. 2021 Nov 16;21:510. doi: 10.1186/s12887-021-02991-8 (PMC8593094; doi:10.1186/s12887-021-02991-8)
Supplement: Supplementary file 1 — Additional file 1: Supplementary Table 1. Changes in Household Environment and Behaviors Reported by Parents at 9–12-month Follow-up of the HSTAP (n = 447). [file 12887_2021_2991_MOESM1_ESM.docx]

**Supplementary Table 1.** Changes in Household Environment and Behaviors Reported by Parents at 9-12-month Follow-up of the HSTAP (n=447)

| **Environment and Behaviors** | **Number (%)** |
| --- | --- |
| Made any change in your household after the education | 386 (86.4) |
| Keep my home free of clutter | 279 (62.4) |
| Do not allow trash to accumulate in my home | 261 (58.4) |
| Do not allow smoking in my home | 249 (55.7) |
| Limit the number of plush toys in my home | 235 (52.6) |
| Monitor and change my air conditioner filters frequently | 224 (50.1) |
| Do not burn candles inside my home | 209 (46.8) |
| Open my windows to ventilate my home | 206 (46.1) |
| Do not use pesticides in my home | 132 (29.5) |
| Keep children away from the stove when cooking | 125 (28.0) |
| Keep pets outdoors | 110 (24.6) |
| Use eco-friendly cleaning products | 103 (23.0) |
| Repair holes in the walls | 62 (13.9) |
| Assure water drains away from my home | 58 (11.2) |
| Repair plumbing leaks | 40 (8.9) |
| Repair peeling or chipped paint in my home | 16 (3.6) |
